# Clinical and microbiological characteristics of bloodstream infection caused by *Klebsiella pneumoniae* harboring *rmpA* in Japanese adults

**DOI:** 10.1038/s41598-023-33265-1

**Published:** 2023-04-21

**Authors:** Shota Kikuchi, Kosuke Kosai, Kenji Ota, Fujiko Mitsumoto-Kaseida, Kei Sakamoto, Hiroo Hasegawa, Koichi Izumikawa, Hiroshi Mukae, Katsunori Yanagihara

**Affiliations:** 1 Department of Laboratory Medicine, Nagasaki University Graduate School of Biomedical Sciences, Nagasaki, Japan; 2 Department of Laboratory Medicine, Nagasaki University Hospital, 1-7-1 Sakamoto, Nagasaki, Nagasaki 852-8501 Japan; 3 Department of Infectious Diseases, Nagasaki University Graduate School of Biomedical Sciences, Nagasaki, Japan; 4 Department of Respiratory Medicine, Nagasaki University Graduate School of Biomedical Sciences, Nagasaki, Japan

**Keywords:** Clinical microbiology, Bacterial infection

## Abstract

We investigated the clinical features of bloodstream infections (BSIs) caused by *Klebsiella pneumoniae* harboring *rmpA* and molecular characteristics of the bacteria. We retrospectively investigated adult patients with *K. pneumoniae* BSI from January 2010 to March 2021 at Nagasaki University Hospital. A matched case–control study in a 1:3 ratio was conducted to clarify the clinical and bacterial characteristics of BSI caused by *rmpA*-positive *K. pneumoniae* compared with those caused by *rmpA*-negative isolates. Antimicrobial susceptibility testing and multilocus sequence typing (MLST) were performed for *rmpA*-positive isolates. The *rmpA* was detected in 36 (13.4%) of the 268 isolates. Of these 36 isolates, 31 (86.1%) harbored *iucA* and 35 (97.2%) each possessed *peg-344* and *iroB*; capsular types were identified as K1 in 9 (25.0%) and K2 in 10 isolates (27.8%). Contrarily, of the 108 *rmpA*-negative isolates, which were matched for case–control studies, 5 isolates (4.6%) harbored *iucA* and 1 (0.9%) each possessed *peg-344* and *iroB*; 2 (1.9%) and 3 isolates (2.8%) had K1 and K2 capsular types, respectively. Among the *rmpA*-positive isolates, ST23/K1 (eight isolates) was the most frequent, followed by ST412/non-K1/K2 (seven isolates), ST86/K2 (five isolates), and ST268/non-K1/K2 (four isolates). In a multivariate analysis using clinical factors, liver abscess positively correlated with *rmpA*-positive isolates, whereas biliary tract infection and use of anticancer drugs negatively correlated with *rmpA*-positive isolates in patients with *K. pneumoniae* BSI. Considering the correlation between *rmpA*-positive isolates and clinical features, *rmpA* can be used as a marker for understanding the pathophysiology of *K. pneumoniae* BSI.

## Introduction

Classical *Klebsiella pneumoniae* causes various infections, such as pneumonia, urinary tract infections, and bacteremia, commonly in hosts with comorbidities^1,2^. However, in recent decades, there have been several reports, primarily from Taiwan, of cases of community-acquired bloodstream infections (BSIs) with liver abscess caused by hypervirulent *K. pneumoniae* (hvKp) in healthy individuals^1–3^, and the spread of the bacterium is a major concern worldwide. Unlike classical *K. pneumoniae*, hvKp causes infections, including meningitis, necrotizing fasciitis, endophthalmitis, and metastatic infections in multiple organs^4^.

Four major virulence factors of *K. pneumoniae* (capsule, lipopolysaccharide, fimbriae, and siderophores) have been reported^5^. Capsules protect the bacterial cells from phagocytosis and antimicrobial peptides and suppress host immunological responses^6–9^. HvKp produces a hypercapsule, which consists of a mucoviscous extracellular polysaccharide; it envelopes the bacterial surface more robustly than a typical capsule. Specific capsular types such as K1 and K2 are associated with increased hvKp pathogenicity^5^.

Plasmid-borne regulator of mucoid phenotype A (*rmpA*) is a transcriptional regulator and enhances capsular polysaccharide synthesis and capsule production^10,11^. Previous studies have shown that the deletion of *rmpA* reduces colony mucoviscosity^10,11^, virulence in mice, and resistance to human serum^10^. Additionally, plasmid-borne *rmpA* has been found to be an accurate marker of hvKp with high sensitivity (0.98) and specificity (0.93)^4^. Therefore, in this study, we focused on *K. pneumoniae* harboring plasmid-borne *rmpA* and investigated the molecular epidemiology and clinical features of BSI caused by the bacterium in our university hospital located in western Japan.

## Materials and methods

### Study design

We retrospectively investigated *K. pneumoniae* isolated from blood samples at Nagasaki University Hospital from January 2010 to March 2021. Adult patients aged 20 years or older, from whose blood samples, *K. pneumoniae* was isolated, were listed from our clinical laboratory database. The first isolate was selected when the bacteria were repeatedly isolated from individual patients during the study^12^. Of the isolates listed, those that were available were included in this study. We collected clinical and microbiological information obtained through routine practice from the medical records and laboratory systems in our hospital. Among patients for whom the isolates were available, a matched case–control study in a 1:3 ratio was conducted to clarify the clinical features of BSI caused by *rmpA*-positive *K. pneumoniae* and the characteristics of the bacterium. Cases and controls were defined as patients from whom *rmpA*-positive and *rmpA*-negative *K. pneumoniae* were isolated, respectively. For case–control matching, age (± 5 years) and sex-matched patients to each case were listed among patients from whom *rmpA*-negative *K. pneumoniae* was isolated, and three patients per case were randomly selected as controls using Microsoft Excel (Microsoft Corporation). The clinical characteristics of the patients with BSI caused by *K. pneumoniae* were compared between the *rmpA*-positive and *rmpA*-negative groups. We evaluated the infection sites with bloodstream infection, such as pneumonia, biliary tract infection, and urinary tract infection, from which *K. pneumoniae* was isolated from each site. Other infection sites, including liver abscess, endophthalmitis, meningitis, and purulent spondylitis, were evaluated regardless of *K. pneumoniae* isolation from each site. The severity of BSI was assessed using the Pitt bacteremia score^13,14^. The study was performed in accordance with tenets of the Declaration of Helsinki and the Ethical Guidelines for Medical and Biological Research Involving Human Subjects. The study protocol including the waiver of consent was approved by the Institutional Review Board of Nagasaki University Hospital (approval number: 21071208).

### Microbiological analysis

Hypermucoviscosity was assessed using the string test, which was considered positive if the viscous string was greater than 5 mm in length when the colony was stretched using a loop on an agar plate^15^. Bacterial DNA was extracted using the boiling method previously described^12^, with minor modifications. Three to five colonies were mixed with 100 µL Tris–EDTA buffer containing 250 U/mL achromopeptidase (Wako Pure Chemical Industries, Ltd.). After incubation at 40 °C for 15 min, 250 µL of 10% Chelex 100 Resin (Bio-Rad) was added, and the mixture was boiled at 99 °C for 5 min, cooled on ice for 1 min, and centrifuged at 12,000 rpm for 1 min. The supernatant was used for the subsequent analyses.

In this study, plasmid-borne *rmpA*, *iucA*, *peg-344*, and *iroB*, which have been reported to be accurate makers of hvKp^4^, as well as capsular types, including K1 (*magA*), K2, and K5, were evaluated using PCR. The PCR primers used were as follows: *rmpA* forward, 5′-ACTGGGCTACCTCTGCTTCA-3′; *rmpA* reverse, 5′-CTTGCATGAGCCATCTTTCA-3′^16,17^; *iucA* forward, 5′-AATCAATGGCTATTCCCGCTG-3′; *iucA* reverse, 5′-CGCTTCACTTCTTTCACTGACAGG-3′^18^; K1 (*magA*) forward, 5′-GGTGCTCTTTACATCATTGC-3′; K1 (*magA*) reverse, 5′-GCAATGGCCATTTGCGTTAG-3′^15^; K2 forward, 5′-GACCCGATATTCATACTTGACAGAG-3′; K2 reverse, 5′-CCTGAAGTAAAATCGTAAATAGATGGC-3′^19^. *peg-344* forward, 5′-CTTGAAACTATCCCTCCAGTC-3′; *peg-344* reverse, 5′-CCAGCGAAAGAATAACCCC-3′^4^; *iroB* forward, 5′-ATCTCATCATCTACCCTCCGCTC-3′; *iroB* reverse, 5′-GGTTCGCCGTCGTTTTCAA-3′^4^; K5 forward, 5′-TGGTAGTGATGCTCGCGA-3′; K5 reverse, 5′-CCTGAACCCACCCCAATC-3′^19^.

DNA was amplified under the following conditions: 5 min at 94 °C, 35 cycles of 30 s at 94 °C, 30 s at the annealing temperature [46 °C for *rmpA*, 50 °C for *iucA*, K1 (*magA*), and K2], and 1 min at 72 °C, and 7 min at 72 °C for the final extension; 10 min at 95 °C, 35 cycles of 30 s at 95 °C, 30 s at the annealing temperature (53 °C for *peg-344* and 59 °C for *iroB*), and 40 s for *peg-344* and 30 s for *iroB* at 72 °C, and 7 min at 72 °C for the final extension; for K5, 1 min at 94 °C, 30 cycles of 30 s at 94 °C, 45 s at 59 °C, and 90 s at 72 °C, and 6 min at 72 °C for the final extension.

Antimicrobial susceptibility was examined using BD Phoenix M50 (Becton Dickinson), according to the manufacturer’s instructions, and determined according to the Clinical and Laboratory Standards Institute (CLSI) M100-Ed33.

Multilocus sequence typing (MLST) was carried out for *rmpA*-positive isolates, based on the sequences of seven housekeeping genes (*gapA*, *infB*, *mdh*, *pgi*, *phoE*, *rpoB*, and *tonB*). The primers used have been described in the *Klebsiella pneumoniae* MLST database (https://bigsdb.pasteur.fr/klebsiella/primers-used/). Direct sequencing was performed as follows. DNA was amplified using primers for each housekeeping gene under the following conditions: 2 min at 94 °C, 35 cycles of 30 s at 94 °C, 1 min at 50 °C, and 30 s at 72 °C, and 5 min at 72 °C for the final extension. The products were purified using a QIA quick PCR purification kit (QIAGEN) or ExoSAP-IT (Applied Biosystems). Fluorescence-based cycle sequencing reactions were performed using the BigDye Terminator v3.1 Cycle Sequencing Kit (Applied Biosystems). After purification using the BigDye Xterminator Purification Kit (Applied Biosystems), the products were analyzed using the SeqStudio Genetic Analyzer (Applied Biosystems). Allele sequences and STs were determined according to the *Klebsiella pneumoniae* MLST database (https://bigsdb.pasteur.fr/klebsiella/).

### Statistical analysis

Numerical variables are expressed as median (interquartile range) and compared using Wilcoxon rank-sum test between groups. Categorical variables were compared using Fisher’s exact test. In the multivariate analysis, variables with P < 0.2 in the univariate analysis were selected and adjusted using the conditional logistic regression model. Data were analyzed using JMP v16 (SAS Institute Inc.), and results with P < 0.05 were considered statistically significant.

## Results

### Microbiological characteristics of *K. pneumoniae* harboring *rmpA*

**Figure 1:**
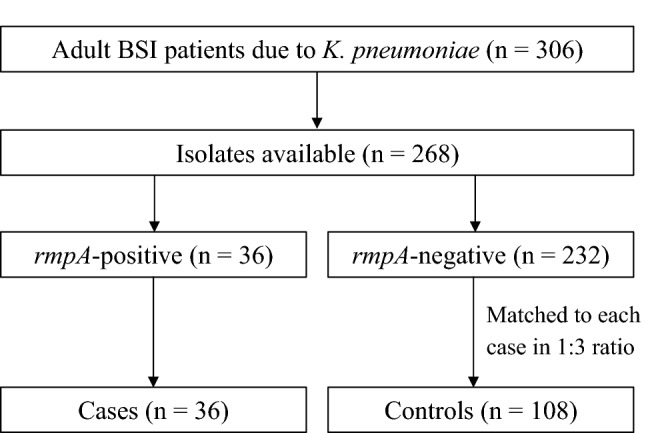
Study design depicting adult patients with bloodstream infection (BSI) and bacterial isolates.

Of the 306 *K. pneumoniae* isolated from the blood of individual patients, 268 were available. Of these 268 isolates, *rmpA* was detected in 36 isolates (13.4%). Of the remaining 232 isolates without *rmpA*, 108 were matched as *rmpA*-negative controls based on the age (± 5 years) and sex of the patients (Fig. 1).

Table 1 shows the characteristics of *rmpA*-positive and *rmpA*-negative isolates. Of the 36 *rmpA*-positive isolates, 31 (86.1%) harbored *iucA* and 35 (97.2%) each possessed *peg-344* and *iroB*. Capsular types were identified as K1 in 9 (25.0%), K2 in 10 (27.8%), and K5 in 1 (2.8%) isolates, respectively. Among the 108 *rmpA*-negative isolates, 5 (4.6%) harbored *iucA* and 1 (0.9%) each possessed *peg-344* and *iroB*; 2 (1.9%), 3 (2.8%), and 1 (0.9%) isolates had K1, K2, and K5 capsular types, respectively. Hyperviscosity was found in 30 *rmpA*-positive isolates (83.3%), which was higher than that in *rmpA*-negative isolates (four isolates, 3.7%).

**Table 1:** Microbiological characteristics of *rmpA*-positive and *rmpA*-negative *K. pneumoniae* isolates from patients with bloodstream infections.

Variable	*rmpA*-positive (n = 36)	*rmpA*-negative (n = 108)	P
Virulence factor			
* iucA*	31 (86.1)	5 (4.6)	< 0.0001
* peg-344*	35 (97.2)	1 (0.9)	< 0.0001
* iroB*	35 (97.2)	1 (0.9)	< 0.0001
Capsular type			
K1 (*magA*)	9 (25.0)	2 (1.9)	< 0.0001
K2	10 (27.8)	3 (2.8)	< 0.0001
K5	1 (2.8)	1 (0.9)	0.4388
String test-positive	30 (83.3)	4 (3.7)	< 0.0001

Data are expressed as number (%).

Table 2 shows the antimicrobial susceptibility of 36 *rmpA*-positive isolates. No carbapenem-resistant isolates and three (8.3%) extended-spectrum β-lactamase (ESBL) producers were identified. Two isolates (5.6%) were resistant to ciprofloxacin.

**Table 2:** Antimicrobial susceptibility of 36 *rmpA*-positive *K. pneumoniae* isolates from patients with bloodstream infections.

Antimicrobial agent	Susceptible	Intermediate	Resistant
Ampicillin	1 (2.8)	8 (22.2)	27 (75.0)
Ampicillin/sulbactam	33 (91.7)	1 (2.8)	2 (5.6)
Piperacillin	32 (88.9)	1 (2.8)^a^	3 (8.3)
Piperacillin/tazobactam	35 (97.2)	0 (0.0)^a^	1 (2.8)
Cefmetazole	36 (100.0)	0 (0.0)	0 (0.0)
Cefotaxime	34 (94.4)	0 (0.0)	2 (5.6)
Ceftriaxone	34 (94.4)	0 (0.0)	2 (5.6)
Ceftazidime	36 (100.0)	0 (0.0)	0 (0.0)
Cefepime	34 (94.4)	1 (2.8)^a^	1 (2.8)
Ceftolozane/tazobactam	35 (97.2)	1 (2.8)	0 (0.0)
Imipenem	33 (91.7)	3 (8.3)	0 (0.0)
Meropenem	36 (100.0)	0 (0.0)	0 (0.0)
Aztreonam	36 (100.0)	0 (0.0)	0 (0.0)
Colistin	–	36 (100.0)	0 (0.0)
Amikacin	34 (94.4)	2 (5.6)	0 (0.0)
Gentamicin	35 (97.2)	0 (0.0)	1 (2.8)
Ciprofloxacin	33 (91.7)	1 (2.8)	2 (5.6)
Levofloxacin	33 (91.7)	3 (8.3)	0 (0.0)
Minocycline	35 (97.2)	1 (2.8)	0 (0.0)
Sulfamethoxazole/trimethoprim	33 (91.7)	0 (0.0)	3 (8.3)

Data are expressed as number (%).

^a^Data are expressed as susceptible-dose dependent (SDD).

Table 3 presents the relationship between MLST and capsular types of the 36 *rmpA*-positive isolates. ST23/K1 (eight isolates) was the most frequent ST/capsular type, followed by ST412/non-K1/K2 (seven isolates), ST86/K2 (five isolates), and ST268/non-K1/K2 (four isolates).

**Table 3:** Relationship between MLST and capsular type of 36 *rmpA*-positive *K. pneumoniae* isolates from patients with bloodstream infections.

MLST	Capsular type	Number of isolates
ST17	K1	1
ST23	K1	8
ST25	K2	1
ST29	Non-K1/K2	1
ST35	Non-K1/K2	1
ST36	Non-K1/K2	1
ST65	K2	3
ST86	K2	5
ST107	Non-K1/K2	1
ST268	Non-K1/K2	4
ST375	K2	1
ST412	Non-K1/K2	7
ST1333	K5	1
ST1764	Non-K1/K2	1

### Clinical features of BSI caused by *K. pneumoniae* harboring *rmpA*

We investigated the baseline characteristics and clinical features of BSI caused by *rmpA*-positive *K. pneumoniae*, compared with those caused by *rmpA*-negative isolates (Table 4). Of the 144 patients analyzed, 91 (63.2%) developed *K. pneumoniae* BSI in the hospital, and the rates were similar between the *rmpA*-positive and *rmpA*-negative groups (63.9% and 63.0%, respectively). The use of anticancer drugs was significantly higher in the *rmpA*-negative group than in the *rmpA*-positive group. Similarly, the presence of malignancy tended to be higher in the *rmpA*-negative group than in the *rmpA*-positive group, but the difference was not significant. Other comorbidities and use of medical devices did not differ between the groups.

**Table 4:** Clinical characteristics of bloodstream infections caused by *rmpA*-positive and *rmpA*-negative *K. pneumoniae*.

Variable	*rmpA*-positive (n = 36)	*rmpA*-negative (n = 108)	P
Age (years)	68.5 (14.5)	69 (13)	–
Sex (male/female)	26/10 (72.2)	78/30 (72.2)	–
Community/hospital	13/23 (36.1)	40/68 (37.0)	1.000
ICU admission	8 (22.2)	15 (13.9)	0.293
Comorbidities/conditions			
Heart disease	11 (30.6)	33 (30.6)	1.000
Pulmonary disease	12 (33.3)	30 (27.8)	0.532
Liver disease	13 (36.1)	48 (44.4)	0.439
Biliary tract disease	4 (11.1)	20 (18.5)	0.439
Renal disease	9 (25.0)	31 (28.7)	0.83
Diabetes mellitus	6 (16.7)	28 (25.9)	0.365
Collagen/autoimmune disease	3 (8.3)	9 (8.3)	1.000
Malignancy	11 (30.6)	54 (50.0)	0.053
Steroids/immunosuppressive agents	7 (19.4)	34 (31.5)	0.204
Anticancer drugs	3 (8.3)	29 (26.9)	0.021
Radiation therapy	1 (2.8)	1 (0.9)	0.439
Endoscopic treatment	2 (5.6)	15 (13.9)	0.241
Surgical procedure	9 (25.0)	21 (19.4)	0.484
Medical devices			
Central venous catheter	6 (16.7)	21 (19.4)	0.809
Tracheal tube	3 (8.3)	9 (8.3)	1.000
Biliary stent/tube	2 (5.6)	15 (13.9)	0.241
Urinary catheter	9 (25.0)	28 (25.9)	1.000
Infection site			
Pneumonia^a^	9 (25.0)	13 (12.0)	0.105
Biliary tract infection^a^	3 (8.3)	30 (27.8)	0.021
Urinary tract infection^a^	8 (22.2)	20 (18.5)	0.632
Liver abscess^b^^,c^	8 (22.2)	8 (7.4)	0.028
Endophthalmitis^b^	0 (0)	1 (0.9)	1.000
Meningitis^b^	1 (2.8)	1 (0.9)	0.439
Purulent spondylitis^b^	1 (2.8)	0 (0)	0.25
Severity			
Pitt bacteremia score	3.5 (4.8)	3 (2)	0.216
Mortality			
7-day	4 (11.1)	4 (3.7)	0.108
28-day	4 (11.1)	11 (10.2)	1.000
In-hospital	6 (16.7)	19 (17.6)	1.000

Data are expressed as median (interquartile range) or number (%).

^a^Clinical diagnosis with *K. pneumoniae* isolation from each site.

^b^Clinical diagnosis regardless of *K. pneumoniae* isolation from each site.

^c^Infectious hepatic cysts in two patients were included.

The biliary tract was the most frequent infection site with BSI (30 patients, 27.8%) in the *rmpA*-negative group, and the rate was higher than that in the *rmpA*-positive group (three patients, 8.3%). Conversely, liver abscess was a more frequent infection in the *rmpA*-positive group (eight patients, 22.2%) than in the *rmpA*-negative group (eight patients, 7.4%). Disease severity assessed using the Pitt bacteremia score was similar, and the mortality rates did not show significant differences between the groups.

Conditional regression analysis was performed to evaluate the correlation between *rmpA*-positive isolates and the clinical factors of patients with *K. pneumoniae* BSI. Variables with P < 0.2 in the univariate analysis (Table 4) were used for the analysis. The presence of liver abscess positively correlated with *rmpA*-positive isolates, whereas biliary tract infection and the use of anticancer drugs showed a negative correlation with *rmpA*-positive isolates in patients with *K. pneumoniae* BSI (Table 5).

**Table 5:** Correlation between *rmpA*-positive isolates and clinical factors in *K. pneumoniae* bloodstream infections.

Variable	OR	95% CI	P
Comorbidities/conditions			
Malignancy	0.816	0.237–2.812	0.748
Anticancer drugs	0.130	0.024–0.715	0.019
Infection site			
Pneumonia^a^	2.177	0.591–8.017	0.242
Biliary tract infection^a^	0.110	0.022–0.558	0.008
Liver abscess^b,c^	8.728	1.729–44.049	0.009
Mortality			
7-day	17.172	0.634–464.914	0.091

OR, odds ratio; CI, confidence interval.

^a^Clinical diagnosis with *K. pneumoniae* isolation from each site.

^b^Clinical diagnosis regardless of *K. pneumoniae* isolation from each site.

^c^Infectious hepatic cysts in two patients were included.

## Discussion

Our study demonstrated the molecular epidemiology of *K. pneumoniae* harboring *rmpA* and the clinical features of BSI caused by the bacterium in our university hospital. Of the 268 *K. pneumoniae* isolates from blood, *rmpA* was detected in 13.4%. After case–control matching (*rmpA*-positive, 36 isolates; *rmpA*-negative, 108 isolates), the positive rates of *iucA*, *peg-344*, and *iroB* were remarkably higher in the *rmpA*-positive group (86.1%, 97.2%, and 97.2%, respectively) than in the *rmpA*-negative group (4.6%, 0.9%, and 0.9%, respectively). In addition to *rmpA*, *iucA*, *peg-344*, and *iroB* have been reported to be accurate markers of hvKp^4^. The high detection rates of these markers in *rmpA*-positive isolates support that *rmpA* is a useful marker of hvKp. Furthermore, K1 and K2 capsular types were identified in 25.0% and 27.8% of the isolates, respectively, in the *rmpA*-positive group, which were clearly higher than those in the *rmpA*-negative group (1.9% and 2.8%, respectively). The STs of *K. pneumoniae* from patients with BSI vary geographically^20^. Our results showed that ST23/K1 was the most prevalent (eight of 36 isolates) in *rmpA*-positive *K. pneumoniae* causing BSI, which is supported by the findings of a previous study on hvKp from Japan^21^. Additionally, we identified ST65/K2 and ST86/K2, similar to that in a previous study in Japan^21^.

This study showed the clinical characteristics of BSI caused by *K. pneumoniae* harboring *rmpA*. Liver abscess was recorded in 22.2% of the patients with BSI in the *rmpA*-positive group, three times more frequently than that in the *rmpA*-negative group (7.4%). The multivariate analysis showed that liver abscess significantly correlated with *rmpA*-positive isolates (odds ratio, 8.728). In addition, all eight *rmpA*-positive *K. pneumoniae* isolates causing liver abscess showed hyperviscosity and carried *iucA*. These results are supported by a recent report that *rmpA*, positive string test, and aerobactin are associated with *K. pneumoniae* causing liver abscess in patients with community-acquired BSI^22^. Furthermore, ST23/K1 (three isolates) and ST65/K2 (two isolates) were identified in five (62.5%) of the eight *rmpA*-positive *K. pneumoniae* isolates that caused liver abscess in our study, which is consistent with previous reports that they are the common ST/capsular types associated with liver abscess in East Asian countries^23–25^. The remaining types that caused liver abscess were ST412/non-K1/K2 (two isolates) and ST268/non-K1/K2 (one isolate).

Meanwhile, the use of anticancer drugs and the presence of biliary tract infection negatively correlated with *rmpA*-positive isolates. Classical *K. pneumoniae* is known to cause bacteremia especially in immunocompromised patients^2^. Therefore, the use of anticancer drugs may reflect the immunocompromised condition of the host. Additionally, biliary tract was a frequent infection site in the *rmpA*-negative group (27.8%) compared with that in the *rmpA*-positive group (8.3%) in this study. A recent study reported a similar result that biliary tract infection was observed more frequently in classical *K. pneumoniae* BSI^26^.

This study has a few limitations. First, as this was a retrospective study, some variables of clinical factors might not have been recorded by attending physicians. Second, the sample size was limited because this study was conducted in a single center, and some isolates were unavailable during the study period. Finally, because we focused on *rmpA*-positive isolates in this study, we could not analyze the microbiological characteristics of *rmpA*-negative isolates in detail.

In conclusion, our study revealed the molecular epidemiology of *K. pneumoniae* harboring *rmpA*, isolated from patients with BSI in our hospital. The presence of *rmpA* correlated with the clinical characteristics of *K. pneumoniae* BSI and can be used as a marker for understanding the pathophysiology of *K. pneumoniae* BSI.

## Supplementary Information

Supplementary Table 1.

## Data Availability

The MLST allele sequences are available in Nagasaki University’s Academic Output Site (http://hdl.handle.net/10069/00041907), and the allele numbers are provided in Supplementary Table 1.
