# Supplementary material for: Clinical and microbiological characteristics of bloodstream infection caused by Klebsiella pneumoniae harboring rmpA in Japanese adults
Source: Sci Rep. 2023 Apr 21;13:6571. doi: 10.1038/s41598-023-33265-1 (PMC10121676; doi:10.1038/s41598-023-33265-1)
Supplement: Supplementary file 1 — Supplementary Table 1. [file 41598_2023_33265_MOESM1_ESM.docx]

Supplementary Table 1 The MLST allele numbers and STs of 36 *rmpA*-positive *K. pneumoniae* isolates from patients with bloodstream infections

| Bacteria | *gapA* | *infB* | *mdh* | *pgi* | *phoE* | *rpoB* | *tonB* | ST |
| --- | --- | --- | --- | --- | --- | --- | --- | --- |
| Kp1  Kp2  Kp3  Kp4  Kp5  Kp6  Kp7  Kp8  Kp9  Kp10  Kp11  Kp12  Kp13  Kp14  Kp15  Kp16  Kp17  Kp18  Kp19  Kp20  Kp21  Kp22  Kp23  Kp24  Kp25  Kp26  Kp27  Kp28  Kp29  Kp30  Kp31  Kp32  Kp33  Kp34  Kp35  Kp36 | 2  2  2  2  9  2  2  2  2  2  2  2  2  2  9  2  9  5  9  2  2  2  2  2  2  2  2  2  2  2  43  2  2  2  9  2 | 1  1  1  1  4  1  1  1  1  1  1  1  3  1  4  1  4  3  4  1  1  1  1  1  1  1  1  1  1  1  1  1  1  1  4  1 | 1  2  2  1  2  1  2  2  2  2  2  1  2  1  2  1  2  1  2  1  1  1  2  1  2  2  2  1  2  2  2  2  2  2  2  2 | 26  1  1  1  1  1  1  1  1  1  1  1  2  1  1  1  1  1  1  1  1  1  17  1  1  1  1  1  1  1  1  1  1  1  1  1 | 12  9  9  9  1  9  9  10  7  7  10  9  6  9  1  9  1  9  1  9  9  4  27  9  7  7  7  10  9  9  10  9  9  10  1  10 | 5  1  1  4  1  4  1  4  1  1  1  4  4  4  1  4  1  4  1  4  4  4  1  4  1  1  1  4  1  1  4  1  1  4  1  4 | 54  112  112  12  27  12  112  13  81  7  19  12  4  12  27  12  27  283  27  12  12  4  39  12  81  81  81  13  112  112  13  112  112  13  27  13 | 1333  412  412  23  86  23  412  65  268  36  35  23  29  23  86  23  86  1764  86  23  23  17  107  23  268  268  268  25  412  412  375  412  412  65  86  65 |
